# Supplementary figures and images for: Compositionally aware estimation of cross-correlations for microbiome data
Source: PLoS One. 2024 Jun 28;19(6):e0305032. doi: 10.1371/journal.pone.0305032 (PMC11213360; doi:10.1371/journal.pone.0305032)

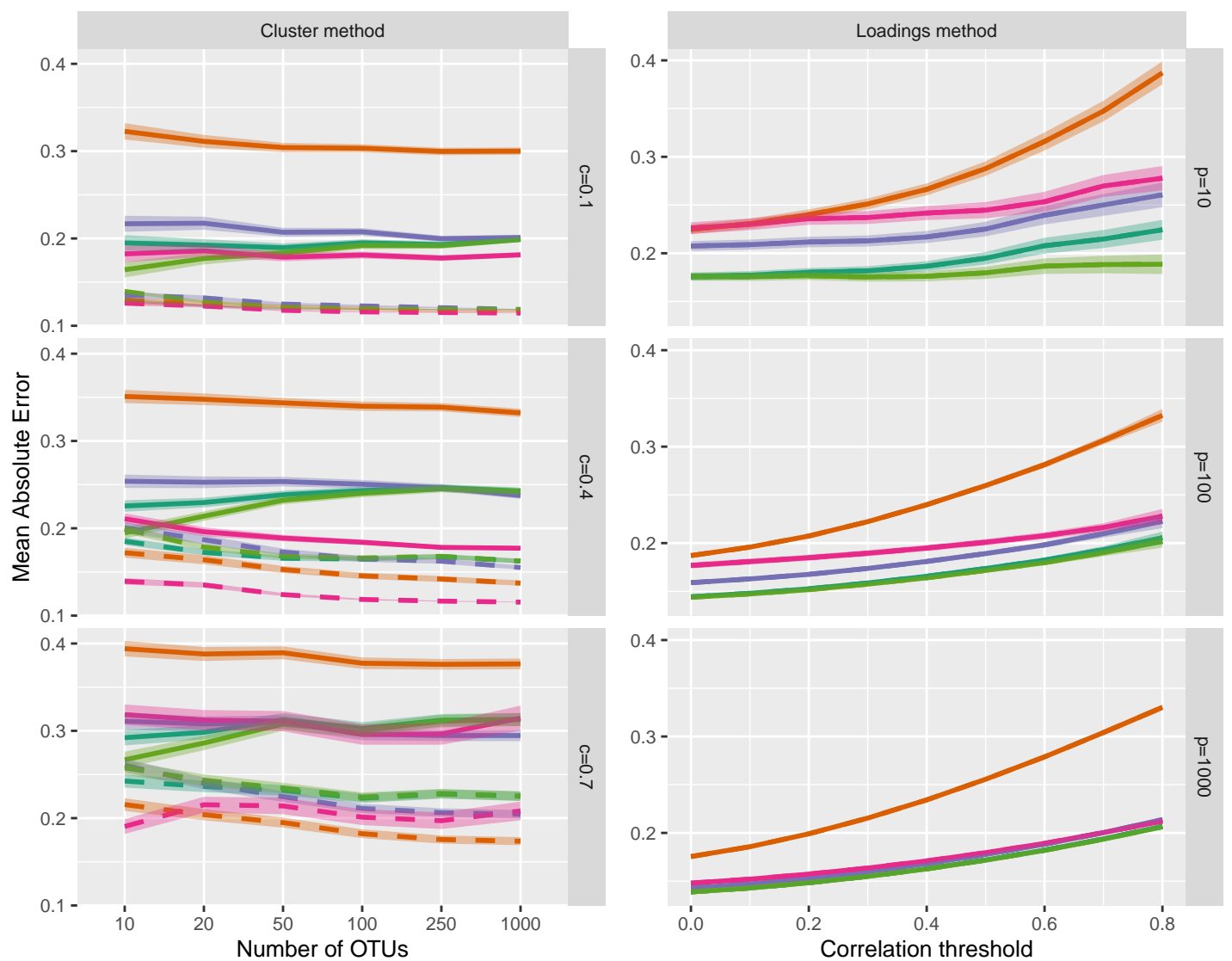

Supplement: S1 Fig — Accuracy of the different cross-correlation methods in case B, in the absence of biological zero by enforcing πj = 0 for j = 1, …, p. Otherwise, the same simulation settings as Fig 1 are used. (PDF) [file pone.0305032.s001.pdf]

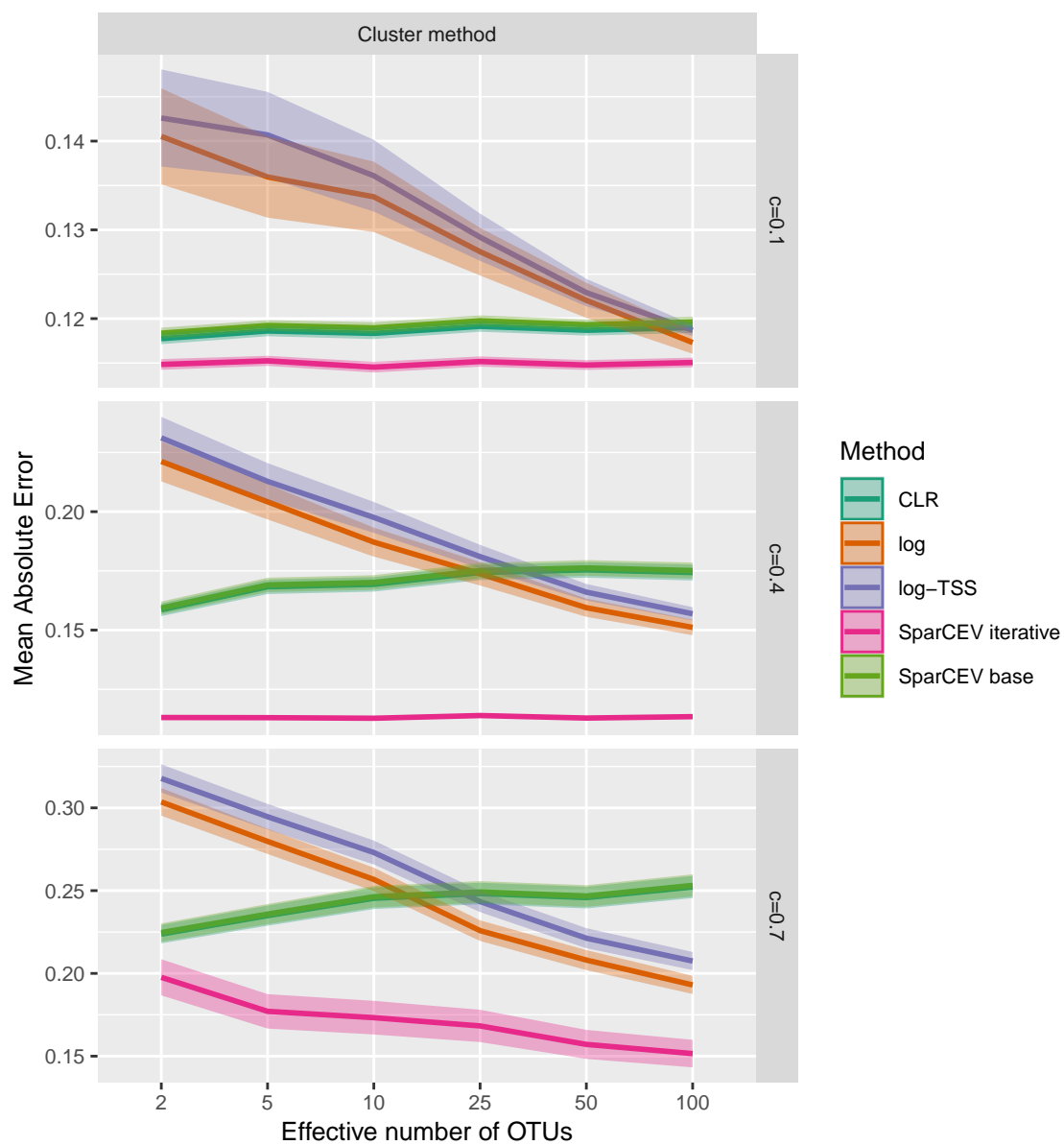

Supplement: S2 Fig — Accuracy of the different cross-correlation methods in case B on uncorrelated pairs at different levels of diversity. (PDF) [file pone.0305032.s002.pdf]

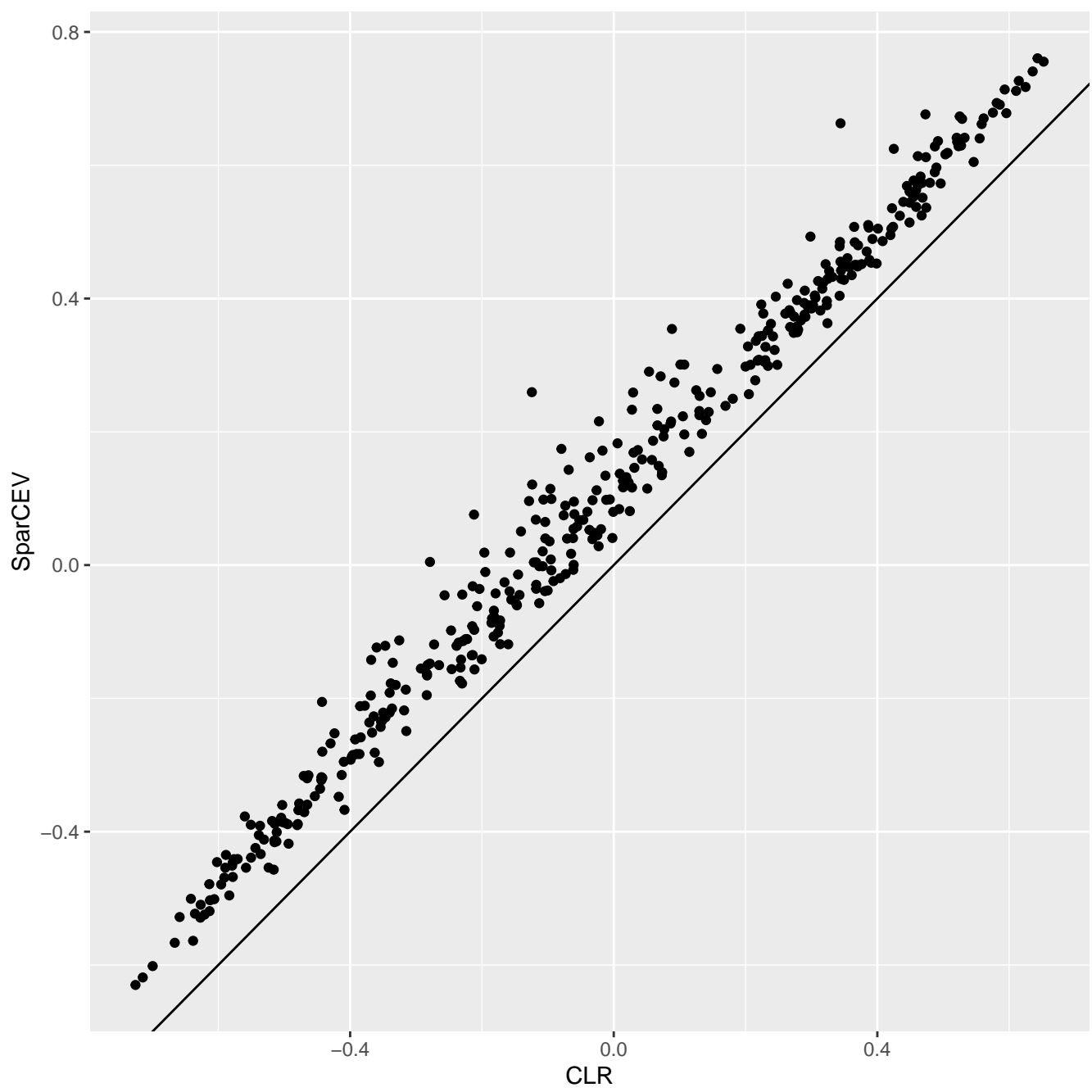

Supplement: S3 Fig — Correlation coefficients estimated by SparCEV and CLR plotted against each other. The straight line has slope 1 and intercept 0. (PDF) [file pone.0305032.s003.pdf]

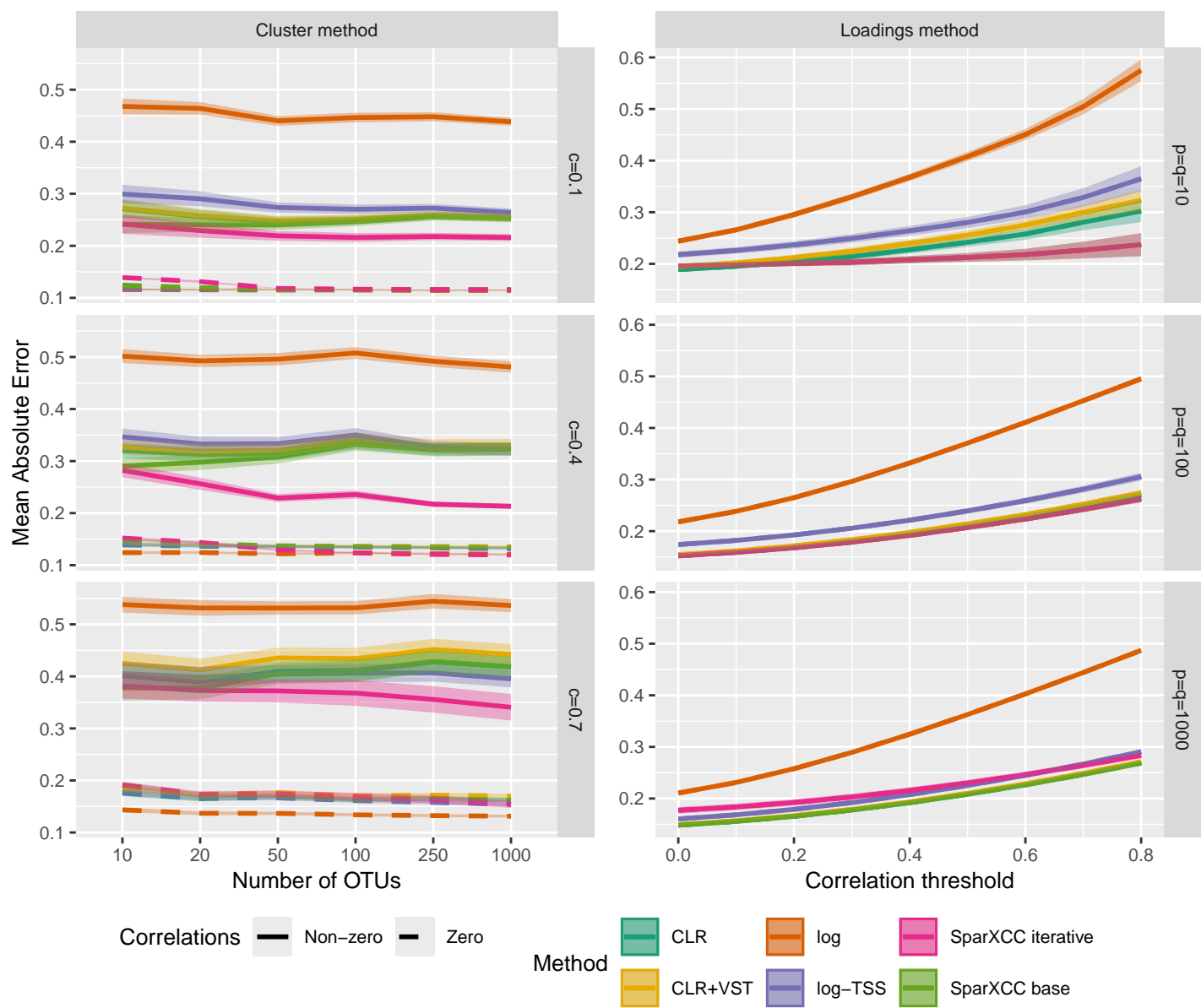

Supplement: S4 Fig — Accuracy of the different cross-correlation methods in case C, in the absence of biological zero by enforcing πj = 0 for j = 1, …, p + q. Otherwise, the same simulation settings as Fig 4 are used. (PDF) [file pone.0305032.s004.pdf]

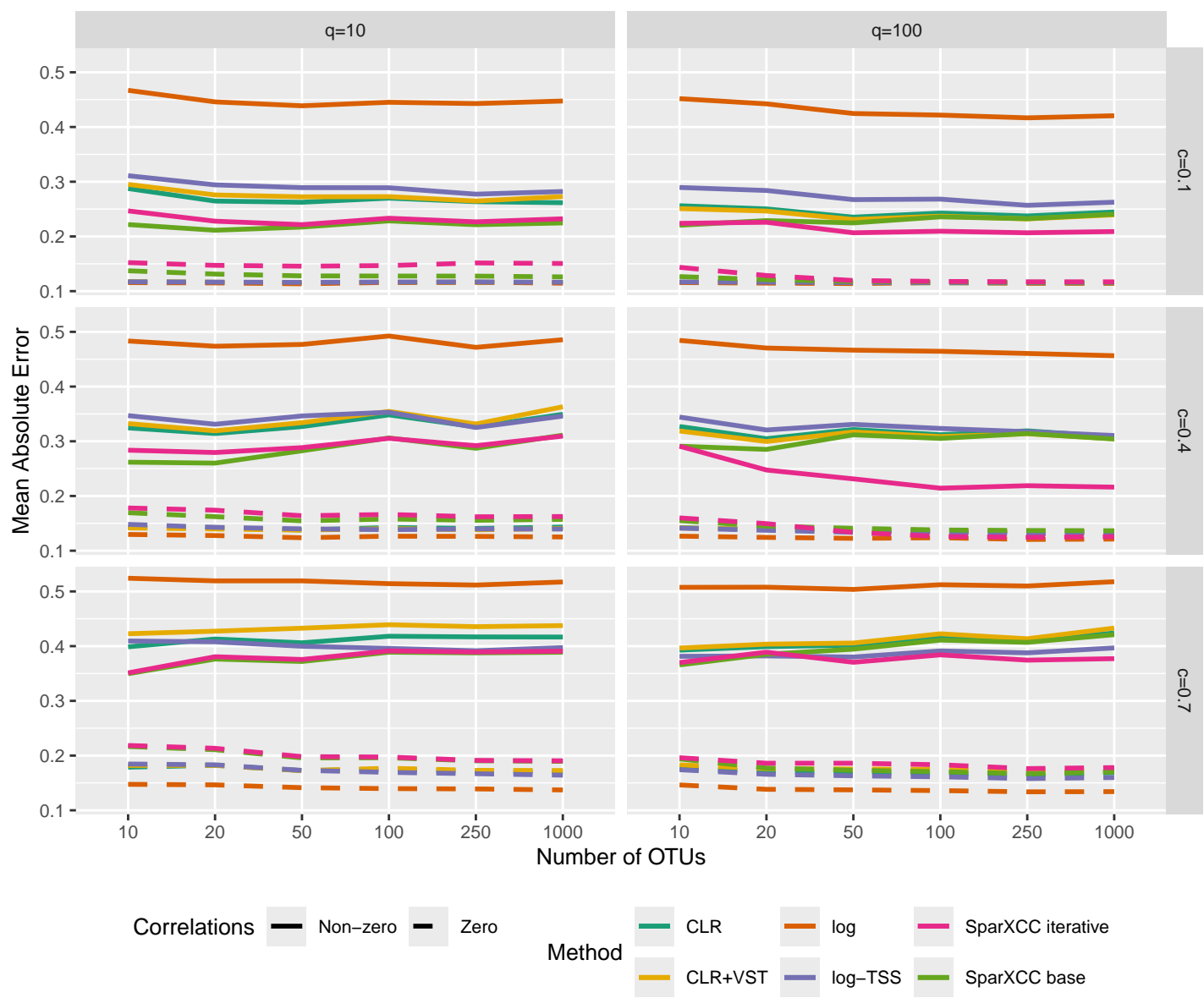

Supplement: S5 Fig — Accuracy of the different cross-correlation methods on correlation matrices generated by the cluster method in case C for q = 10, 100. Otherwise, the same simulation settings as Fig 4 are used. (PDF) [file pone.0305032.s005.pdf]

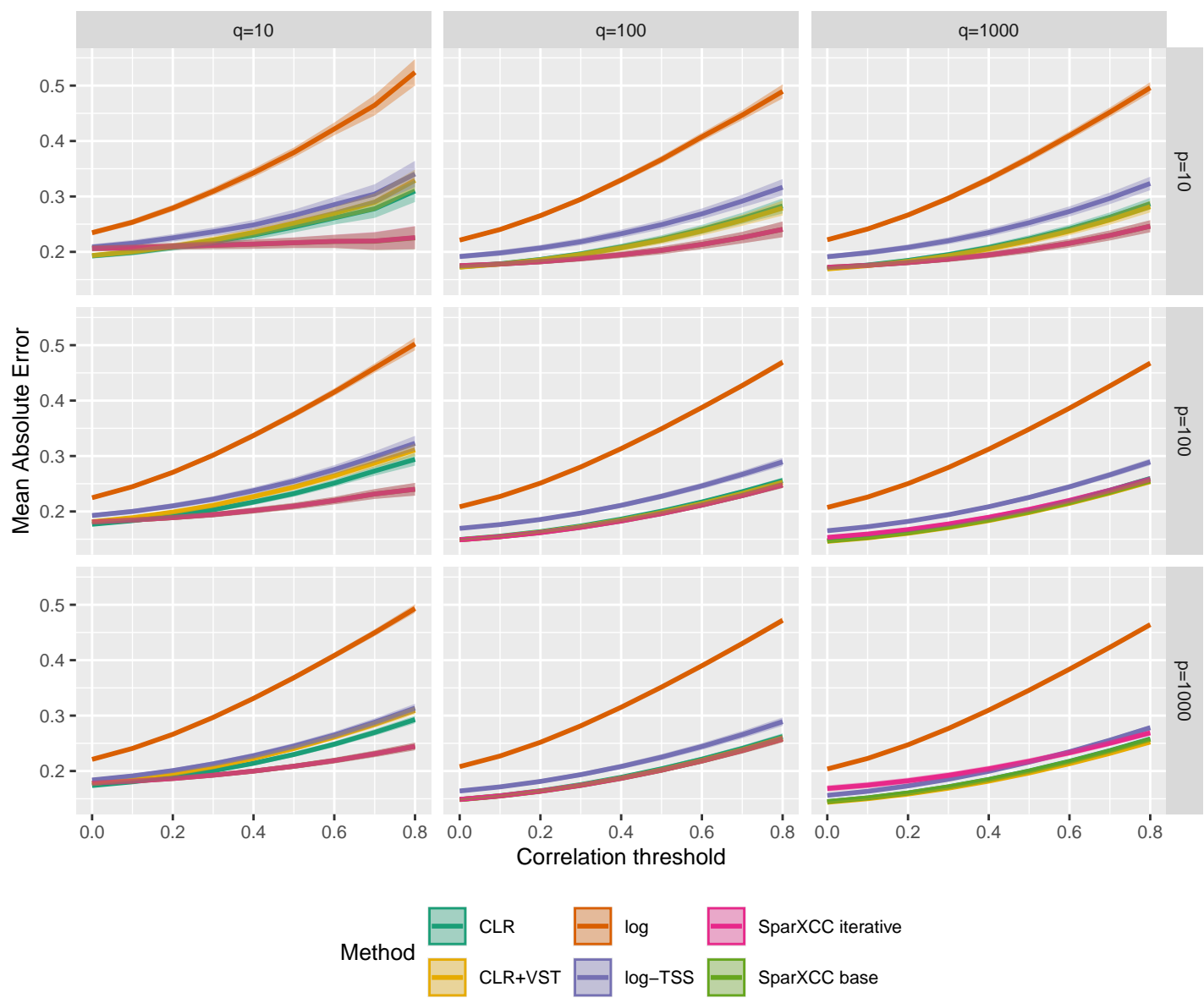

Supplement: S6 Fig — Accuracy of the different cross-correlation methods on correlation matrices generated by the loadings method in case C for all combinations of p = 10, 100, 1000 and q = 10, 100, 1000. Otherwise, the same simulation settings as Fig 4 are used. (PDF) [file pone.0305032.s006.pdf]

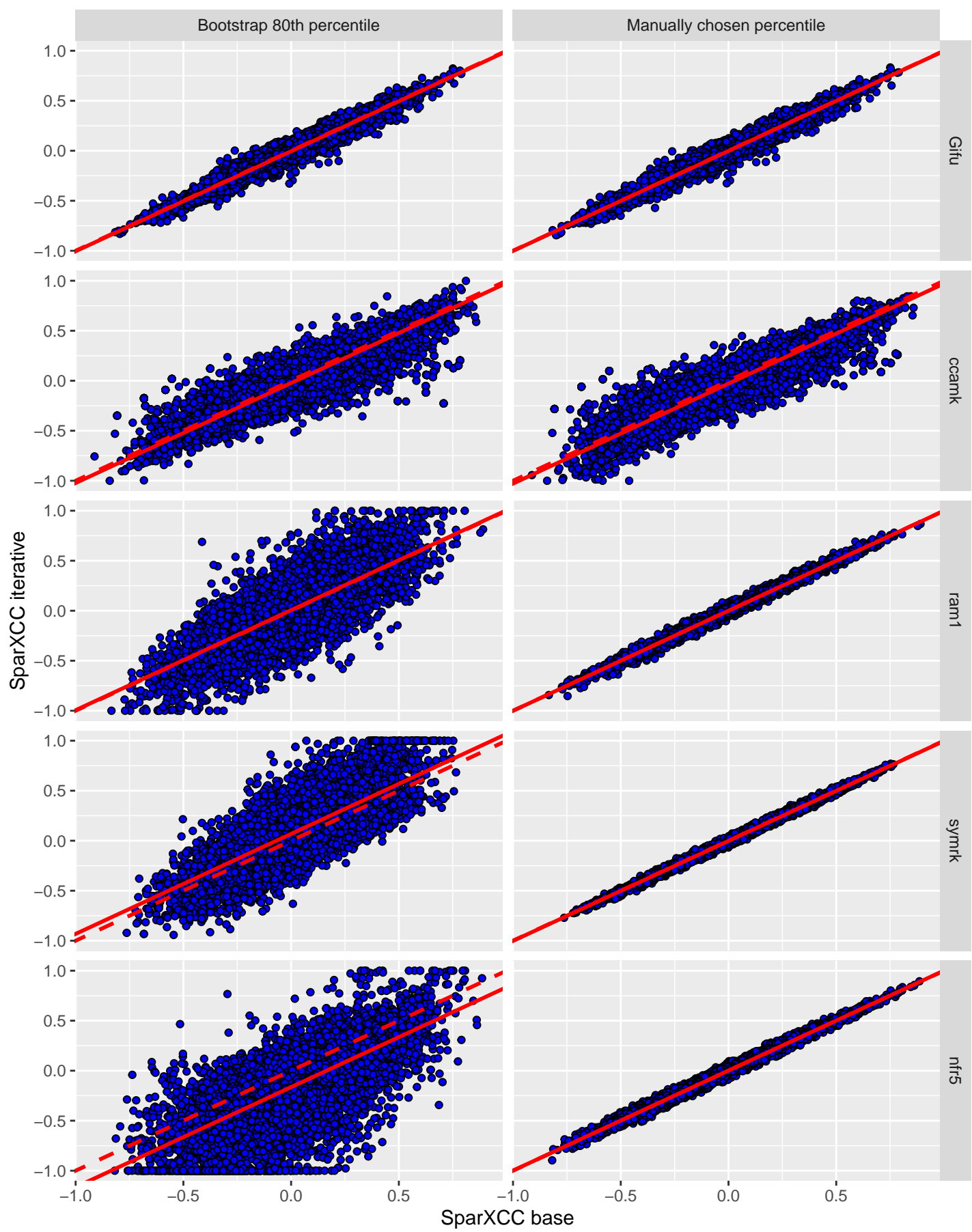

Supplement: S7 Fig — The correlation coefficients estimated by SparXCC base and SparXCC iterative plotted against each other for both the default choice of threshold (the 80th percentile) and a threshold chosen after manually evaluating percentiles of the permutations. (PDF) [file pone.0305032.s007.pdf]

**Gifu**

***symrk***

***nfr5***

***ram1***

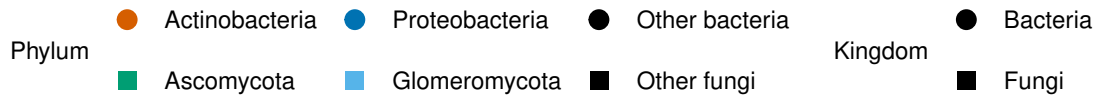

Supplement: S8 Fig — Graph with edges between nodes when the cross-correlation is above a permutation threshold, estimated by SparXCC on rhizosphere data. (PDF) [file pone.0305032.s008.pdf]

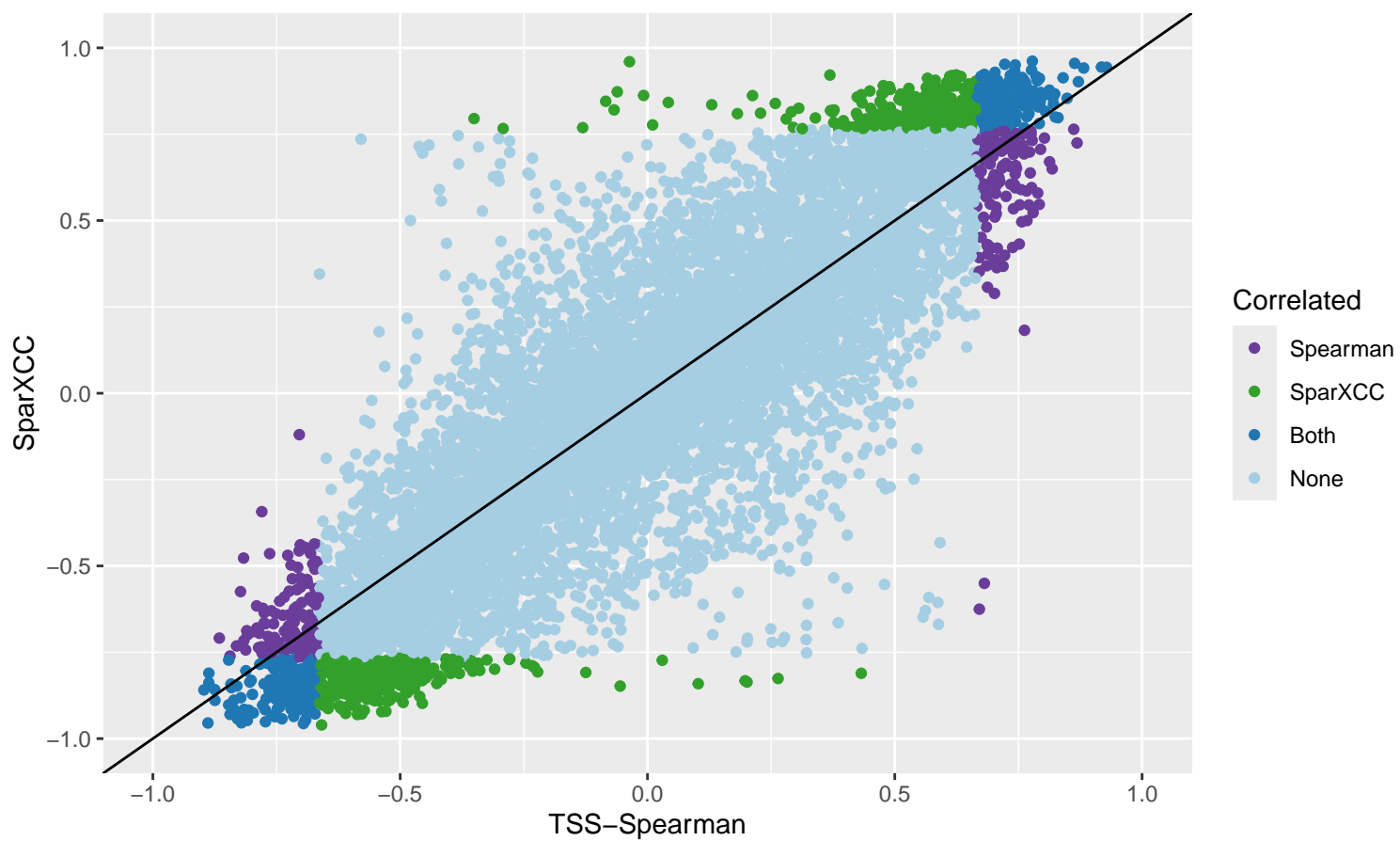

Supplement: S9 Fig — The estimated correlation coefficients as estimated by Spearman correlations of relative abundances plotted against correlations approximated by SparXCC. For Spearman, a pair is considered correlated when a t-test returns a p-value less than 0.001. For SparXCC, a pair is considered correlated when it is above the permutation threshold. (PDF) [file pone.0305032.s009.pdf]

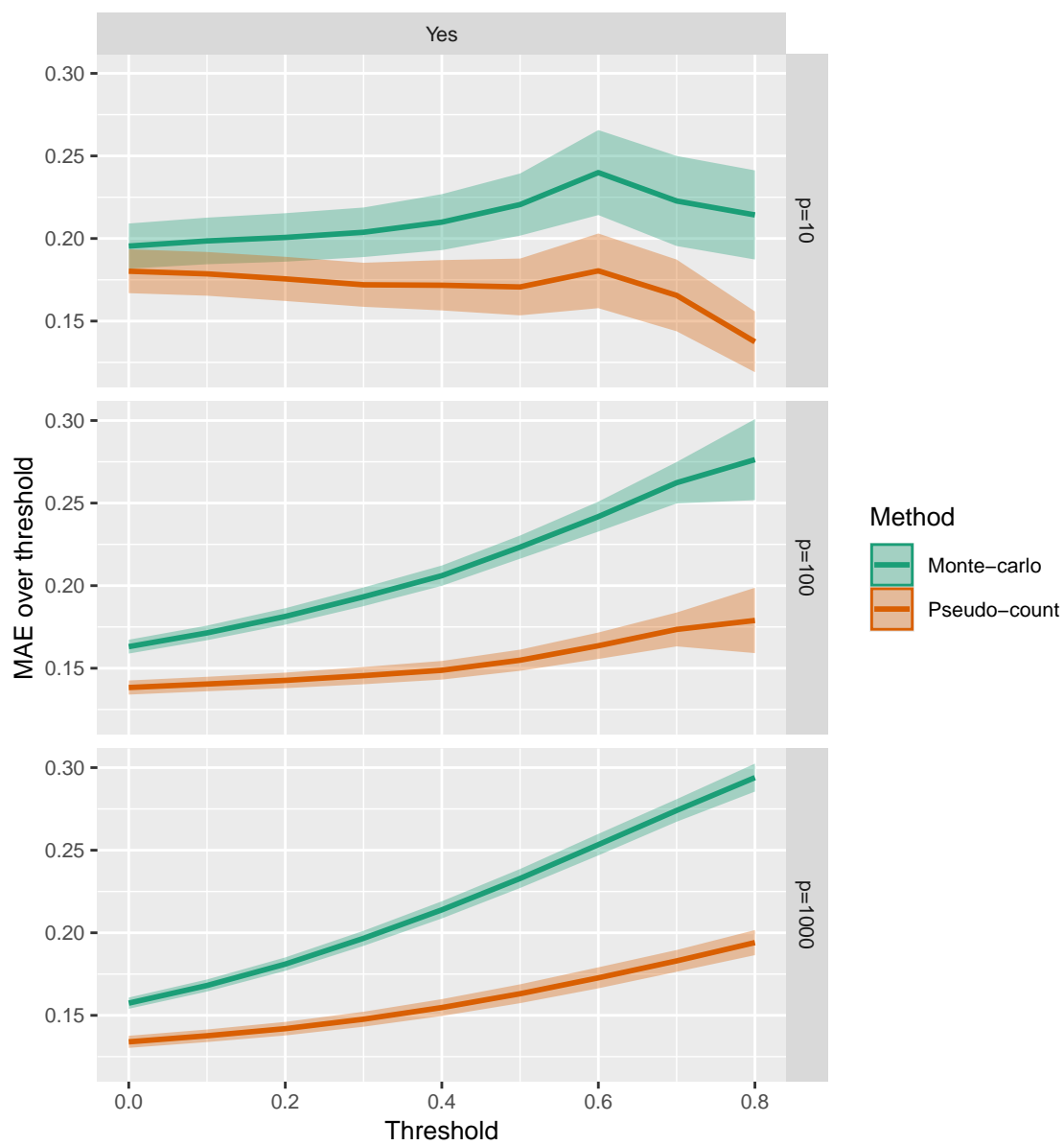

Supplement: S10 Fig — Accuracy of using a pseudo-count versus Dirichlet Monte Carlo for SparCEV. (PDF) [file pone.0305032.s010.pdf]

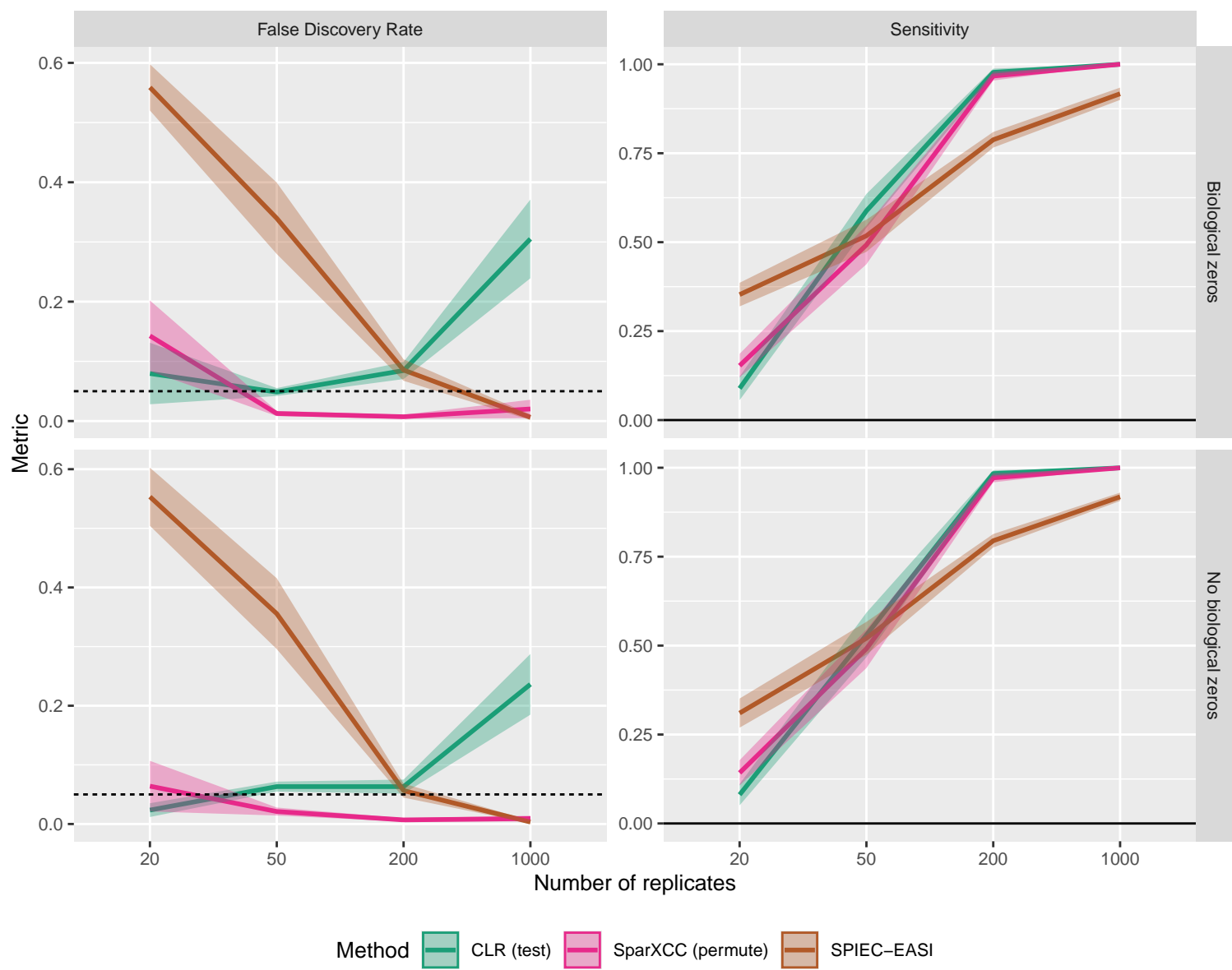

Supplement: S11 Fig — Power and FDR of CLR with a t-test (p- values corrected for multiple testing with Benjamini-Hochberg), SparXCC with permutation thresholding, and SPIEC-EASI. (PDF) [file pone.0305032.s011.pdf]

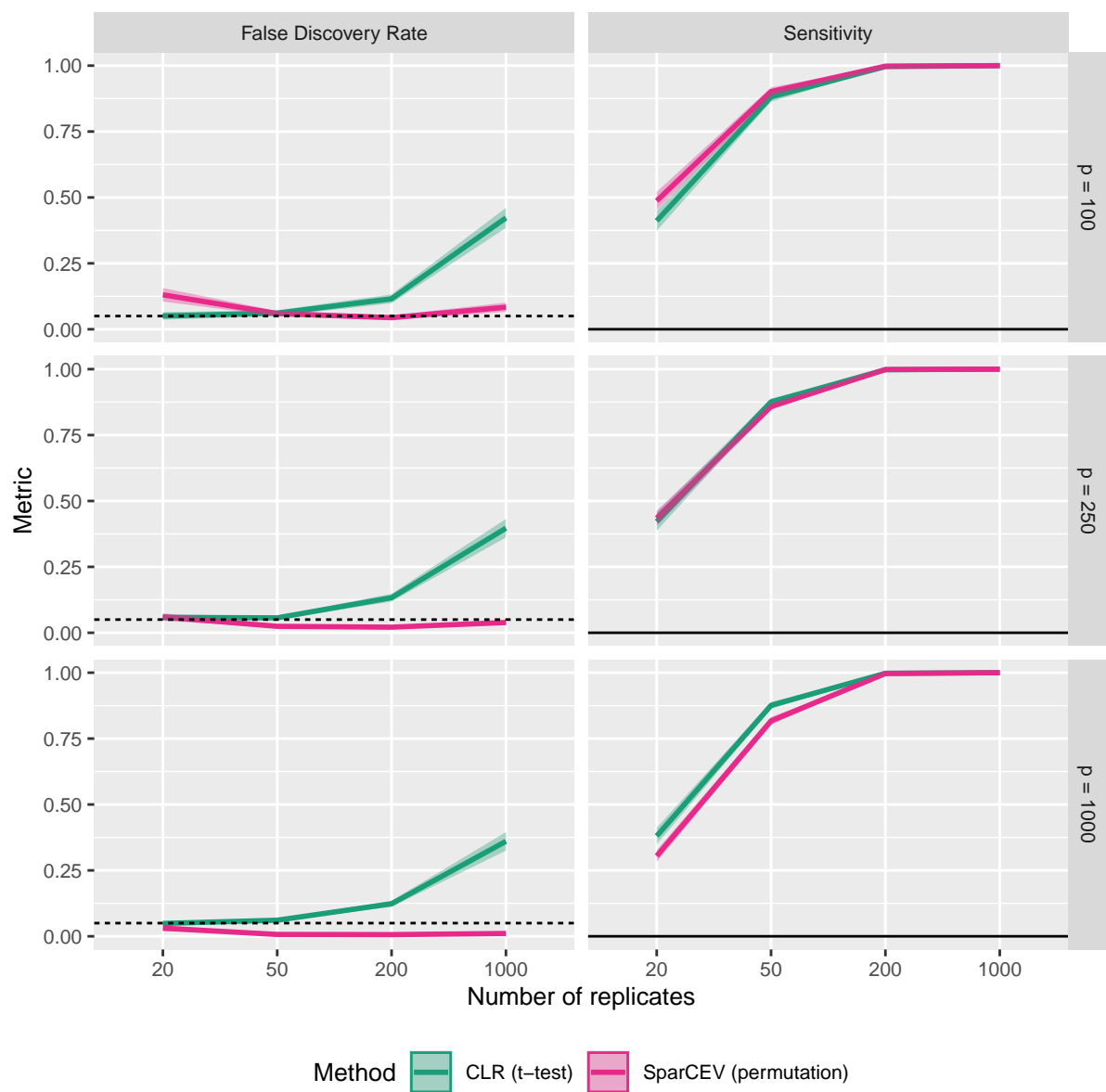

Supplement: S12 Fig — Power and FDR of CLR with a t-test (p- values corrected for multiple testing with Benjamini-Hochberg) and SparCEV with permutation thresholding. (PDF) [file pone.0305032.s012.pdf]
