# Supplementary material for: Compositionally aware estimation of cross-correlations for microbiome data
Source: PLoS One. 2024 Jun 28;19(6):e0305032. doi: 10.1371/journal.pone.0305032 (PMC11213360; doi:10.1371/journal.pone.0305032)
Supplement: S1 Text — (PDF) [file pone.0305032.s017.pdf]

Compositionally aware estimation of cross-correlations for microbiome data: supplementary text

Ib Thorsgaard Jensen[12*], Luc Janss[3], Simona Radutoui[1], Rasmus Waagepetersen[2**],

1 Department of Molecular Biology and Genetics, Aarhus University, Denmark
3 Department of Mathematical Sciences, Aalborg University, Denmark
2 Center for Quantitative Genetics and Genomics, Aarhus University, 8000 Aarhus, Denmark

* itj@math.aau.dk
** rw@math.aau.dk

Throughout this supplementary note, we use the same notation as in the main text of the paper. We derive the methods SparCEV and SparXCC for estimation of cross-correlations for compositional datasets, inspired by Friedman and Alm [1].

1 Transformation-based Correlation Approximations

We have

$$\mathbb{C}\text{ov}\left[\log x_i, b\right] = \mathbb{C}\text{ov}\left[\log a_i, b\right] - \mathbb{C}\text{ov}\left[\log A, b\right] + \mathbb{C}\text{ov}\left[\log N, b\right] \approx \mathbb{C}\text{ov}\left[\log a_i, b\right],$$

provided the phenotype variable is approximately uncorrelated with the library size and the total bacterial load. With similar assumptions, we have

$$\mathbb{C}\text{ov}\left[\log \text{TSS}(x_i), b\right] = \mathbb{C}\text{ov}\left[\log a_i, b\right] - \mathbb{C}\text{ov}\left[\log A, b\right] \approx \mathbb{C}\text{ov}\left[\log a_i, b\right].$$

Finally, we also have

$$\begin{aligned}\mathbb{C}\text{ov}\left[\text{CLR}(x_i), b\right] = \mathbb{C}\text{ov}\left[\text{CLR}(a_i), b\right] &= \mathbb{C}\text{ov}\left[\log a_i, b\right] - \frac{1}{p}\sum_{j=1}^{p}\mathbb{C}\text{ov}\left[\log a_j, b\right] \\ &\approx \mathbb{C}\text{ov}\left[\log a_i, b\right],\end{aligned}$$

which holds, when $\frac{1}{p}\sum_{j=1}^{p}\mathbb{C}\text{ov}\left[\log a_j, b\right] \approx 0$. Thus, we see that all approaches can approximate $\mathbb{C}\text{ov}\left[\log a_i, b\right]$ under appropriate assumptions. For the variance, we have

$$\begin{aligned}\mathbb{V}\text{ar}\left[\log x_i\right] &= \mathbb{V}\text{ar}\left[\log a_i\right] + \mathbb{V}\text{ar}\left[\log A\right] - 2\mathbb{C}\text{ov}\left[\log a_i, \log A\right] + \mathbb{V}\text{ar}\left[\log N\right] \\ &\approx \mathbb{V}\text{ar}\left[\log a_i\right] + \mathbb{V}\text{ar}\left[\log A\right] + \mathbb{V}\text{ar}\left[\log N\right].\end{aligned}$$

Note that the above approximation holds when $\mathbb{C}\text{ov}\left[\log a_i, \log A\right] \approx 0$. This may be violated, if OTU i makes up a sufficiently large proportion of the microbiome or if it is strongly correlated with many other OTUs. Regardless, we cannot assume that $\mathbb{V}\text{ar}\left[\log A\right]$ or $\mathbb{V}\text{ar}\left[\log N\right]$ are small, and thus we do not get a good approximation of $\mathbb{V}\text{ar}\left[\log a_i\right]$ using log-transformed read-counts. We have

$$\begin{aligned}\mathbb{V}\text{ar}\left[\log \text{TSS}(x_i)\right] &= \mathbb{V}\text{ar}\left[\log a_i\right] + \mathbb{V}\text{ar}\left[\log A\right] - 2\mathbb{C}\text{ov}\left[\log a_i, \log A\right] \\ &\approx \mathbb{V}\text{ar}\left[\log a_i\right] + \mathbb{V}\text{ar}\left[\log A\right],\end{aligned}$$

and thus we do not get a good approximation of $\mathbb{V}\text{ar}\left[\log a_i\right]$ from log-TSS either. Finally, we have,

$$\begin{aligned}\mathbb{V}\text{ar}\left[\text{CLR}(x_i)\right] &= \mathbb{V}\text{ar}\left[\log a_i\right] + \mathbb{V}\text{ar}\left[\frac{1}{p}\sum_{j=1}^{p}\log a_j\right] - 2\mathbb{C}\text{ov}\left[\log a_i, \frac{1}{p}\sum_{j=1}^{p}\log a_j\right] \\ &= \mathbb{V}\text{ar}\left[\log a_i\right] + \frac{1}{p^2}\sum_{j=1}^{p}\sum_{k=1}^{p}\mathbb{C}\text{ov}\left[\log a_j, \log a_k\right] - \frac{1}{p}\sum_{j=1}^{p}2\mathbb{C}\text{ov}\left[\log a_i, \log a_j\right] \\ &\approx \frac{p-2}{p}\mathbb{V}\text{ar}\left[\log a_i\right] + \frac{1}{p^2}\sum_{j=1}^{p}\mathbb{V}\text{ar}\left[\log a_j\right] \approx \mathbb{V}\text{ar}\left[\log a_i\right],\end{aligned}$$

where the last relation holds when p is large, and the second to last relation hold when

$$\frac{1}{p^2}\sum_{k=1}^{p}\sum_{j\neq k}\mathbb{C}\mathrm{ov}\left[\log a_j, \log a_k\right] \approx 0, \quad \frac{1}{p}\sum_{j\neq i}\mathbb{C}\mathrm{ov}\left[\log a_i, \log a_j\right] \approx 0.$$

2 Derivation of SparCEV

Suppose $x_i = a_i/\sum_j a_j$ for absolute abundances $a_1,\ldots,a_p$ and that y is some scalar nominal variable. Then,

$$\mathbb{C}\mathrm{ov}\left[\log\frac{x_i}{x_j}, y\right] = \mathbb{C}\mathrm{ov}\left[\log\frac{a_i}{a_j}, y\right] = \mathbb{C}\mathrm{ov}\left[\log a_i, y\right] - \mathbb{C}\mathrm{ov}\left[\log a_j, y\right].$$

Thus

$$\sum_{j\neq i}\mathbb{C}\mathrm{ov}\left[\log\frac{x_i}{x_j}, y\right] = (p-1)\left(\mathbb{C}\mathrm{ov}\left[\log a_i, y\right] - \frac{1}{p-1}\sum_{j\neq i}\mathbb{C}\mathrm{ov}\left[\log a_j, y\right]\right) \approx (p-1)\mathbb{C}\mathrm{ov}\left[\log a_i, y\right],$$

where the approximation comes from the assumption that $\frac{1}{p-1}\sum_{j\neq i}\mathbb{C}\mathrm{ov}\left[\log a_j, y\right] \approx 0$. Thus we get

$$\mathbb{C}\mathrm{orr}\left[\log a_i, y\right] \approx \frac{1}{\sigma_y \alpha_i}\frac{1}{p-1}\sum_{j\neq i}\mathbb{C}\mathrm{ov}\left[\log\frac{x_i}{x_j}, y\right],$$

where $\sigma_y^2 = \mathbb{V}\mathrm{ar}\left[y\right]$, and $\alpha_i^2 = \mathbb{V}\mathrm{ar}\left[\log a_i\right]$. The variance σ_y^2 can be estimated in a standard fashion and α_i^2 can be estimated using SparCC assuming that most abundances are not correlated.

Suppose we have $R = \{i : \mathbb{C}\mathrm{orr}\left[\log a_i, y\right] = 0\}$. Then,

$$\sum_{j\in R}\mathbb{C}\mathrm{ov}\left[\log\frac{x_i}{x_j}, y\right] = |R|\left(\mathbb{C}\mathrm{ov}\left[\log a_i, y\right] - \frac{1}{|R|}\sum_{j\in R}\mathbb{C}\mathrm{ov}\left[\log a_j, y\right]\right) = |R|\mathbb{C}\mathrm{ov}\left[\log a_i, y\right].$$

Thus, we get the exact relation

$$\mathbb{C}\mathrm{orr}\left[\log a_i, y\right] = \frac{1}{|R|\sigma_y \alpha_i}\sum_{j\in R}\mathbb{C}\mathrm{ov}\left[\log\frac{x_i}{x_j}, y\right],$$

which motivates the iterative procedure described in the main text.

3 Derivation of SparXCC

Suppose $y_k = b_k/\sum_l b_l$ for absolute expression levels $b_1,\ldots,b_q$ in addition to x_i and a_i defined as above for $i = 1,\ldots,p$. Now, define

$$t_{ijkl} = \mathbb{C}\mathrm{ov}\left[\log\frac{x_i}{x_j}, \log\frac{y_k}{y_l}\right] = \mathbb{C}\mathrm{ov}\left[\log\frac{a_i}{a_k}, \log\frac{b_l}{b_l}\right].$$

Then,

$$\begin{aligned}
t_{ik} :=& \sum_{j=1}^{p}\sum_{l=1}^{q}\mathbb{C}\mathrm{ov}\left[\log\frac{a_i}{a_k}, \log\frac{b_l}{b_l}\right] = \sum_{j\neq i}\sum_{l\neq k}\mathbb{C}\mathrm{ov}\left[\log\frac{a_i}{a_k}, \log\frac{b_l}{b_l}\right] \\
&= \sum_{j\neq i}\sum_{l\neq k}\Big(\mathbb{C}\mathrm{ov}\left[\log a_i, \log b_k\right] - \mathbb{C}\mathrm{ov}\left[\log a_j, \log b_k\right] \\
&- \mathbb{C}\mathrm{ov}\left[\log a_i, \log b_l\right] + \mathbb{C}\mathrm{ov}\left[\log a_j, \log b_l\right]\Big) \\
&= (p-1)(q-1)\mathbb{C}\mathrm{ov}\left[\log a_i, \log b_k\right] - (q-1)\sum_{j\neq i}\mathbb{C}\mathrm{ov}\left[\log a_j, \log b_k\right] \\
&- (p-1)\sum_{l\neq k}\mathbb{C}\mathrm{ov}\left[\log a_i, \log b_l\right] + \sum_{j\neq i}\sum_{l\neq k}\mathbb{C}\mathrm{ov}\left[\log a_j, \log b_l\right] \\
&\approx (p-1)(q-1)\mathbb{C}\mathrm{ov}\left[\log a_i, \log b_k\right] = (p-1)(q-1)\alpha_i\beta_k\rho_{ik},
\end{aligned}$$

where $\beta_k^2 = \mathbb{V}\text{ar}[\log b_k]$ and the approximation follows from the assumption that the last three terms are small. By re-arranging this, we obtain

$$\rho_{ik} \approx \frac{t_{ik}}{(p-1)(q-1)\alpha_i\beta_k}. \tag{1}$$

Thus, we estimate

$$\hat{\rho}_{ik} = \frac{\hat{t}_{ik}}{(p-1)(q-1)\hat{\alpha}_i\hat{\beta}_k}. \tag{2}$$

Let $S = \{i : \rho_{ik} = 0 \text{ for all } k\}$ and $T = \{k : \rho_{ik} = 0 \text{ for all } i\}$. Then,

$$\begin{aligned}\sum_{j\in S}\sum_{l\in T}\mathbb{C}\text{ov}\left[\log\frac{a_i}{a_k}, \log\frac{b_l}{b_l}\right] &= \sum_{j\in R}\sum_{l\in T}\Big(\mathbb{C}\text{ov}[\log a_i, \log b_k] - \mathbb{C}\text{ov}[\log a_j, \log b_k] \\ &\qquad - \mathbb{C}\text{ov}[\log a_i, \log b_l] + \mathbb{C}\text{ov}[\log a_j, \log b_l]\Big) \\ &= |S||T|\mathbb{C}\text{ov}[\log a_i, \log b_k] = |S||T|\alpha_i\beta_k\rho_{ik},\end{aligned}$$

which motivates the iterative procedure in the main text.

Efficient Estimation of Variance Sums

Exploiting symmetry $t_{ijkl} = t_{jilk}$ and that $t_{iikk} = 0$, a naive approach to estimating the t_{ik}s involves estimation of $pq(pq-1)/2$ covariances $t_{ijkl} = \mathbb{C}\text{ov}\left[\log\frac{x_i}{y_k}, \log\frac{x_j}{y_l}\right]$. In practice this may be not be feasible since p and q are often fairly large, e.g $p = 5779$ and $q = 3360$ in the genotype data analyzed in the paper. We would then need to estimate approximately 188 trillion covariances. Using 32-bit number representations this would take up 685 TB of memory and be prohibitively time consuming. Fortunately it is possible to estimate the t_{ik}s without estimating each term in the sum. To reduce the number of covariances, can exploit the fact that

$$t_{ik} = \sum_{j=1}^{p}\sum_{l=1}^{q}\mathbb{C}\text{ov}\left[\log\frac{x_i}{x_j}, \log\frac{y_k}{y_l}\right] = \mathbb{C}\text{ov}\left[p\log x_i - \sum_{j=1}^{p}\log x_j, q\log y_k - \sum_{l=1}^{q}\log y_l\right],$$

and thus only pq covariances need to be estimated.

4 Construction of correlation matrices

We consider two approaches to construct the correlation matrix R needed for the simulation scheme detailed above.

Cluster Method

Suppose we have OTU abundances $a_1, \ldots, a_p$ and other variables $b_{p+1}, \ldots, b_{p+q}$. A correlation matrix R is constructed as follows.

1. Pick a proportion $0 < c < 1$.
2. Randomly select a set of indices, $M_a \subseteq \{1, 2, \ldots, p\}$ containing $\max\{\lfloor cp\rfloor, 1\}$ indices and a set $M_b \subseteq \{1, 2, \ldots, q\}$ containing $\max\{\lfloor cq\rfloor, 1\}$ indices.
3. Simulate $c^+ \sim \text{Unif}(0, 1)$
4. Let M_a^+ be a set of $\lfloor c^+|M_a|\rfloor$ indices, sampled without replacement from M_a and let $M_a^- = M_a \setminus M_a^+$. Construct M_b^+ and M_b^- in the same way.
5. Let $M = M_a \cup M_b$, $M^+ = M_a^+ \cup M_b^+$ and $M^- = M_a^- \cup M_b^-$.

6. Pick a correlation strength, $\rho \in (0, 1)$.

7. Construct R with ones on the diagonal and off-diagonal entries according to Table 1.

It is easy to check that the generated R is positive definite. The interpretation of the procedure above is that we construct a cluster of OTUs and a cluster of other variables. Non-zero positive or negative correlation exists within and between clusters. Variables outside the clusters are not correlated with any other variables. This produces a simple correlation matrix where it is easy to control the level of sparsity and the strength of the correlations.

	$j \in M^+$	$j \in M^-$	$j \notin M$
$i \in M^+$	ρ	$-\rho$	0
$i \in M^-$	$-\rho$	ρ	0
$i \notin M$	0	0	0

Table 1: Off-diagonal entries R_{ij} of the matrix constructed with the cluster method.

Loadings Method

In addition to the cluster method, we also employ a procedure that does not produce zero correlations but where correlations are generally small with a few outlying large correlations.

1. Choose some $k < p + q$ and generate a $p + q \times k$ matrix Q with independent random entries (here generated from a standard normal distribution).

2. Let $U = QQ^\top$. U is symmetric and positive semi-definite, but rank-deficient.

3. Generate a $p + q \times p + q$ diagonal matrix with a positive diagonal (here generated as absolute values of independent standard normal variables).

4. Let $C = U + D$. C is a symmetric full-rank positive definite matrix, and thus the covariance matrix of a non-degenerate distribution.

5. Let $R = E^{-1/2} C E^{-1/2}$, where $E = \text{diag}\{C_{11}, \ldots, C_{p+q,p+q}\}$.

The above procedure does not guarantee that the sparsity assumption holds. However, in practice we find that the average correlations are typically close to 0, provided $p + q$ is not too small. For large k the correlations are more concentrated around 0. Since we want presence of highly correlated pairs of variables we consider $k = 5$.

References

[1] Friedman J, Alm EJ. Inferring Correlation Networks from Genomic Survey Data. PLoS Computational Biology. 2012;8(9):e1002687. doi:10.1371/journal.pcbi.1002687.
