# Supplementary material for: Compositionally aware estimation of cross-correlations for microbiome data
Source: PLoS One. 2024 Jun 28;19(6):e0305032. doi: 10.1371/journal.pone.0305032 (PMC11213360; doi:10.1371/journal.pone.0305032)
Supplement: Attachment — Submitted filename: Response to reviwers.pdf [file pone.0305032.s018.pdf]

Response to the editor

We thank the editor for constructive feedback on the formatting and practical aspects of the paper. In the following we address their concerns.

Editor Point 1 — When submitting your revision, we need you to address these additional requirements. Please ensure that your manuscript meets PLOS ONE's style requirements, including those for file naming. The PLOS ONE style templates can be found at https://journals.plos.org/plosone/s/file?id=wjVg/PLOSOne_formatting_sample_main_body.pdf and https://journals.plos.org/plosone/s/file?id=ba62/PLOSOne_formatting_sample_title_authors_affiliations.pdf.

Reply: The manuscript has been edited to comply with the PLOS One style guidelines. Specifically, headings has been changed to sentence case and their font sizes has been adjusted, the main text has been changed to double space paragraph format, and the "Support information" section has been moved down after the references. Additionally, addresses have been removed from author affiliations and changes in author affiliations since the time of the original submission have been incorporated.

Editor Point 2 — Please note that PLOS ONE has specific guidelines on code sharing for submissions in which author-generated code underpins the findings in the manuscript. In these cases, all author-generated code must be made available without restrictions upon publication of the work. Please review our guidelines at
https://journals.plos.org/plosone/s/materials-and-software-sharing#loc-sharing-code
and ensure that your code is shared in a way that follows best practice and facilitates reproducibility and reuse.

Reply: Implementations of the methods SparCEV and SparXCC are now available as an R-package at https://github.com/IbTJensen/CompoCor. Everything else necessary to replicate the results of the paper is available at https://github.com/IbTJensen/Microbiome-Cross-correlations.

Editor Point 3 — Note from Emily Chenette, Editor in Chief of PLOS ONE, and Iain Hrynaszkiewicz, Director of Open Research Solutions at PLOS: Did you know that depositing data in a repository is associated with up to a 25% citation advantage (https://doi.org/10.1371/journal.pone.0230416)? If you've not already done so, consider depositing your raw data in a repository to ensure your work is read, appreciated and cited by the largest possible audience. You'll also earn an Accessible Data icon on your published paper if you deposit your data in any participating repository (https://plos.org/open-science/open-data/#accessible-data).

Reply: See reply to Editor Point 2.

Editor Point 4 — Thank you for stating the following in the Acknowledgments Section of your manuscript:

[Funding: This work was supported by the Bill and Melinda Gates Foundation and from Foreign, Commonwealth & Development Office through Engineering the Nitrogen Symbiosis for Africa (ENSA; OPP11772165). We thank Adri´an G´omez Repoll´es for assistance with the dermatitis

data. We thank Thorsten Thiergart and Ruben Garrido-Oter for assistance with the plant microbiome data. We thank B Kirtley Amos and Max Gordon for critical reading. We thank Sha Zhang for supplying the data used to construct the templates for gene expression data in the simulation studies. We thank Taylor Grace FitzGerald for copy-editing]

We note that you have provided funding information that is not currently declared in your Funding Statement. However, funding information should not appear in the Acknowledgments section or other areas of your manuscript. We will only publish funding information present in the Funding Statement section of the online submission form. Please remove any funding-related text from the manuscript and let us know how you would like to update your Funding Statement. Currently, your Funding Statement reads as follows: [This work was supported by the Bill and Melinda Gates Foundation and from Foreign, Commonwealth & Development Office through Engineering the Nitrogen Symbiosis for Africa (ENSA; OPP11772165). The funders played no role in the content of this paper.] Please include your amended statements within your cover letter; we will change the online submission form on your behalf.

Reply: We have removed the funding information from the Acknowledgments section in the manuscript. Please add the following to the funding statement:
"Ib Thorsgaard Jensen and Rasmus Waagepetersen were supported by research grant VIL57389 from Villum Fonden."

Response to the reviewers

We thank the reviewers for their critical assessment of our work. In the following we address their concerns point by point.

Reviewer 1

Reviewer Point 1 — In the section analyzing real-world data on atopic dermatitis, it would be highly beneficial if the authors could present the highly correlated microbial species identified by other methods. A detailed comparison, particularly focusing on overlaps and distinctions among these methodologies, would greatly enhance our understanding of the uniqueness and effectiveness of your proposed approach.

Reply: We have now added a more thorough discussion on the differences between the results found by SparCEV, CLR and log-TSS. Additionally, a supplementary table provides the results with all three methods on all families (this table was also included in the previous version, but it was not mentioned in the text. This has now been rectified.).

Reviewer Point 2 — The dynamic threshold selection method introduced in the article seems to rely significantly on subjective judgment, such as the user-defined parameter 't'. This reliance might impact the reproducibility and objectivity of the results. I would recommend that the authors

explore this method in more depth, providing a more stable criterion for dynamic threshold selection or more objective guidelines to augment the universality and reliability of the method.

Reply: Inspired by this comment, we made a refinement to the estimation procedures. Specifically, we implemented an iterative procedure similar to the one utilized by SparCC, which both SparCEV and SparXCC are based on. We had initially written this off after initially seeing poor results, but after more thorough investigation, we found that it can help alleviate the bias caused by the sparsity assumption. This obviates the need for the parameter *t*. We have rerun all the simulation studies and included this new iterative approach. In some cases we see a substantial gain in accuracy, while in others we see a decrease in accuracy (in cases where the sparsity assumption is almost exact). We also provide practical guidance for assessing whether or not the iterative procedure is appropriate on a given dataset.

The iterative procedure makes use of user-specified thresholds, t, t_1, and t_2 to select "weakly correlated OTUs/genes" (no connection to the t from the previous version of the manuscript). However, we believe these are of a different nature than the t from the previous version of the manuscript for the following reasons: Firstly, SparCC, which is already widely used in the microbiome literature, uses a similar procedure with a similar user-specified parameter. Secondly, we suggest a bootstrap approach to select them in a data-driven way. Thirdly, we suggest a diagnostic plot to assess whether or not SparCEV/SparXCC with the iterative procedure provides an improvement over SparCEV/SparXCC without it on a given dataset. In contrast, the t from the previous version of the manuscript could neither be selected nor evaluated for a specific dataset.

Reviewer Point 3 — Given that the manuscript indicates similar outcomes for SparXCC and CLR transformations in Case C with large p and q values, albeit being time-consuming, a comparative analysis of the computational time across different methods as a function of data scale would be instructive. Such analysis would aid in assessing the efficiency and applicability of these methods in practical scenarios.

Reply: An analysis of the running time is now included in the manuscript. Additionally, we explored a different approach for the mathematical derivation of SparXCC. A different way to express the covariances was formulated, and it was easily shown to be equivalent to the formulation from the previous manuscript. Using this formulation substantially speeds up SparXCC. Additionally, with the iterative procedure SparXCC can provide substantially better results than CLR in some cases, even when p and q are large, which we believe justifies the greater running time.

Reviewer Point 4 — In the process of distinguishing between correlated and uncorrelated pairs, a t-test has been applied to CLR. I would urge the authors to clearly specify the exact correction method used for addressing multiple comparisons. For example, was a Bonferroni correction or a Benjamini-Hochberg procedure employed? Clarity in this aspect is crucial for assessing the statistical rigor of the study.

Reply: Throughout the manuscript, all *p*-values are corrected for multiple testing with Benjamini-Hochberg (except in the plant microbiome data example, where we follow the method employed in the original paper for the purposes of comparison). This has now been clearly indicated in every instance.

Additional changes to manuscript

- Due to the addition of the iterative procedure to SparCEV and SparXCC, all figures had to be updated.
- The abstract, results, discussion, and conclusion has been changed to incorporate results obtained when using the iterative versions of SparCEV and SparXCC.
- The description of SparXCC in materials and methods has been changed to the more efficient formulation mentioned in the reply to Reviewer Point 3. The derivation in S1 text has likewise been changed to this formulation.
- Material and methods now describe the iterative procedures.
- When selecting the threshold m, we now use the maximum instead of the $1 - 1/(pq)$ quantile. The results found in the plant microbiome data were updated as a result.
- In the analysis of the atopic dermatitis dataset, SparCEV with the iterative procedure is now used, and the results are updated accordingly. In connection with the more detailed comparison with other methods requested by the reviewer, an additional supplementary figure was added comparing the estimates by CLR and SparCEV. Additionally, the results from SparCEV base and SparCEV iterative are compared.
- In the analysis, we compare SparXCC base and SparXCC iterative and conclude that SparXCC base is more appropriate on this dataset. In connection to this, an additional supplementary figure is added.

We thank both the reviewer and the editor for constructive feedback, and we look forward to hearing back regarding our revised manuscript.

Sincerely,

Ib Thorsgaard Jensen, PhD,
Department of Mathematical Sciences,
University of Aalborg,
Skjernvej 4A, 9220 Aalborg Øst, Denmark
itj@math.aau.dk

Professor Rasmus Waagepetersen, PhD,
Department of Mathematical Sciences,
University of Aalborg,
Skjernvej 4A, 9220 Aalborg Øst, Denmark
rw@math.aau.dk
